# Comparative Endothelial Cell Response on Topographically Patterned Titanium and Silicon Substrates with Micrometer to Sub-Micrometer Feature Sizes

**DOI:** 10.1371/journal.pone.0111465

**Published:** 2014-10-30

**Authors:** Prashanthi Vandrangi, Shannon C. Gott, Ryan Kozaka, Victor G. J. Rodgers, Masaru P. Rao

**Affiliations:** 1 Department of Mechanical Engineering, University of California Riverside, Riverside, California, United States of America; 2 Department of Bioengineering, University of California Riverside, Riverside, California, United States of America; 3 Materials Science and Engineering Program, University of California Riverside, Riverside, California, United States of America; Universita' degli Studi del Salento, Italy

## Abstract

In this work, we evaluate the *in vitro* response of endothelial cells (EC) to variation in precisely-defined, micrometer to sub-micrometer scale topography on two different substrate materials, titanium (Ti) and silicon (Si). Both substrates possess identically-patterned surfaces composed of microfabricated, groove-based gratings with groove widths ranging from 0.5 to 50 µm, grating pitch twice the groove width, and groove depth of 1.3 µm. These specific materials are chosen due to their relevance for implantable microdevice applications, while grating-based patterns are chosen for the potential they afford for inducing elongated and aligned cellular morphologies reminiscent of the native endothelium. Using EA926 cells, a human EC variant, we show significant improvement in cellular adhesion, proliferation, morphology, and function with decreasing feature size on patterned Ti substrates. Moreover, we show similar trending on patterned Si substrates, albeit to a lesser extent than on comparably patterned Ti substrates. Collectively, these results suggest promise for sub-micrometer topographic patterning in general, and sub-micrometer patterning of Ti specifically, as a means for enhancing endothelialization and neovascularisation for novel implantable microdevice applications.

## Introduction

It is now well-known that modulation of substrate topography within the micro- to nanoscale regime can profoundly influence cell-substrate interactions [1]–[8]. Moreover, it is also well-known that surfaces with precisely-defined periodic topographies (hereafter referred to as “patterned” surfaces) can exert stronger influence than randomly-structured surfaces of comparable morphological length scales (e.g., surfaces with stochastic nanoscale roughness produced by acid etching, thin-film deposition, cold nanoparticle compaction, etc.) [1]–[3], [5]. Finally, it is becoming increasingly clear that anisotropically patterned surfaces (e.g., groove-based gratings) afford opportunity for inducing desirable cellular responses (e.g., elongation and alignment) that are not possible with more isotropic surface patterns (e.g., hole or post arrays) [1]–[3], [5]. Consequently, this suggests that micro- to nanoscale anisotropic patterning may eventually provide a powerful new means for modulating cellular response to implantable devices.

One area of particular interest in this regard has been the modulation of endothelial cell (EC) response to polymeric vascular graft materials, where the ultimate goal has been to facilitate endothelialization over the graft surface and minimize cellular detachment. A number of recent *in* *vitro* studies have demonstrated that patterning of such materials with micrometer to sub-micrometer scale gratings can favorably affect EC responses such as adhesion, proliferation, and morphology, among others [9]–[25]. This, therefore, suggests promise for enhancing endothelialization *in* *vivo*. However, understanding of EC response to other patterned materials, such as titanium (Ti) and silicon (Si), is limited. This represents an important knowledge gap, since patterning may provide means for enhancing the performance of other novel implantable microdevices based upon these materials (e.g., Ti-based pro-healing vascular stents [26], or Si-based wirelessly-controlled implantable drug delivery microchips [27]).

Herein, we begin to address this knowledge gap through the study of *in* *vitro* EC response on Ti and Si substrates with identically-patterned surfaces composed of groove-based gratings with groove widths ranging from 0.5 to 50 µm, grating pitch twice the groove width, and groove depth of 1.3 µm. Fabrication of these precisely-defined patterned surfaces is enabled by both conventional Si micromachining techniques, as well as our novel Ti Deep Reactive Ion Etching (Ti DRIE) process [28], [29], which provides opportunity for machining of Ti at length scales well beneath that which is possible with other techniques (e.g., laser micromachining, microelectrodischarge machining, ultraprecision CNC, etc.).

Using EA926 cells, a human EC variant, we show that cellular responses such as adhesion, proliferation, elongation, and athero-resistant signaling are enhanced with decreasing pattern feature size for both materials; however, the magnitude of these responses is considerably larger on patterned Ti relative to comparably patterned Si. Collectively, these results suggest promise for sub-micrometer patterning of Ti as a new means for enhancing endothelialization and neovascularisation for novel implantable microdevice applications.

## Materials and Methods

### Patterned Substrate Design

**Figure 1:**
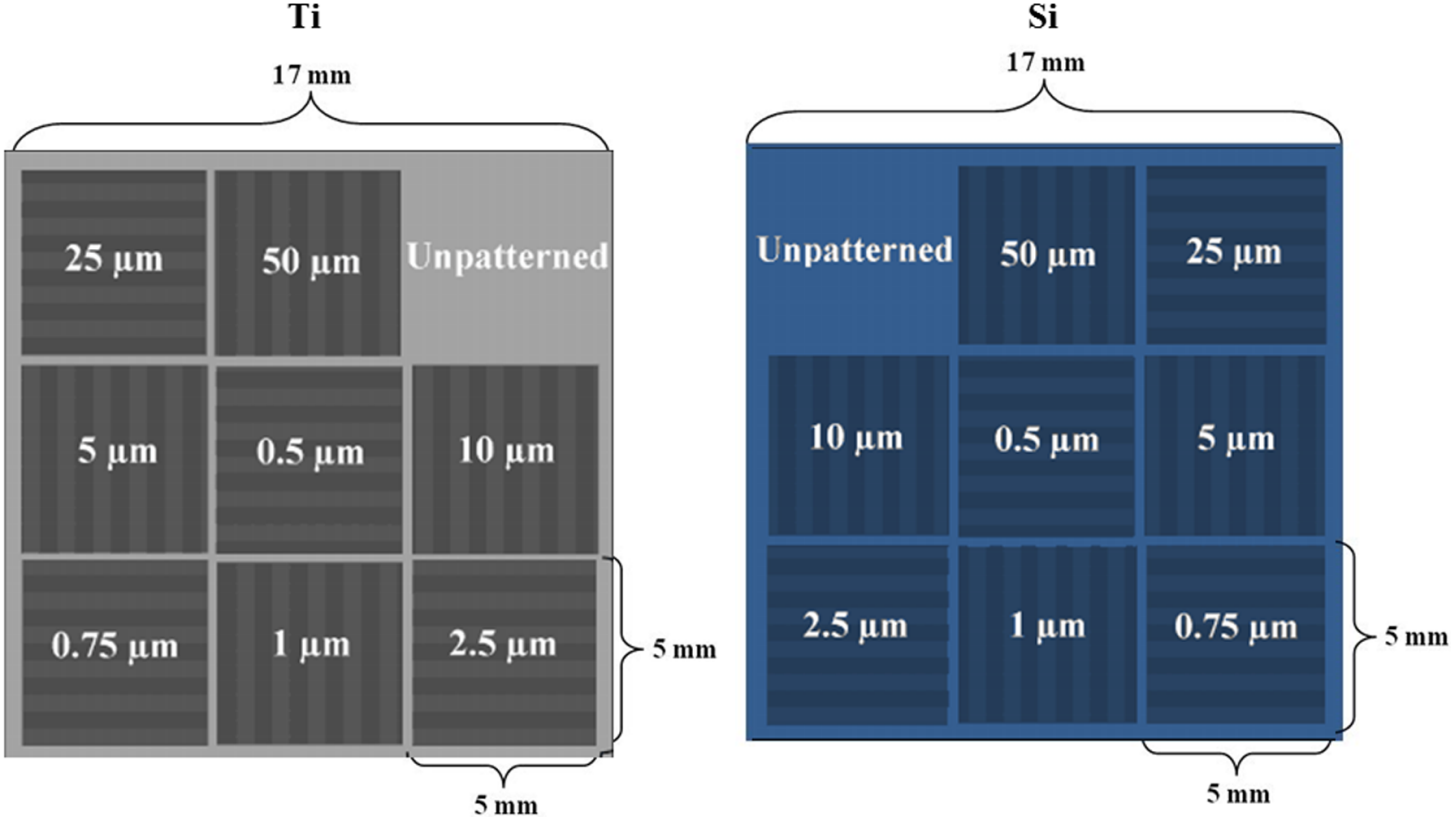
Schematics of the patterned Ti (left) and Si (right) substrates used in the current study. Each substrate is composed of an array of nine sub-patterns, one of which is left unpatterned to serve as a control, and the other eight of which consist of groove-based gratings with groove widths indicated in the schematic.

Figure 1 schematically illustrates the layouts of the patterned Ti and Si substrates used in this study, both of which share identical dimensions and patterning. One of the sub-patterns in each substrate is left unpatterned as a control, while the remainder are surface gratings consisting of periodic groove arrays with groove widths ranging from 0.5 to 50 µm, and grating pitch equal to twice the groove width (i.e., grating pitch = groove width + ridge width). Each grating sub-pattern is orthogonally-oriented with respect to its neighbors, and is surrounded by a 100 µm wide unpatterned border (thus yielding 200 µm total width of unpatterned region between neighboring sub-patterns). Use of this substrate layout provides opportunity for simultaneous evaluation of a broad feature size range within the same substrate, and therefore, within the same cell culture conditions.

### Patterned Substrate Fabrication

**Figure 2:**
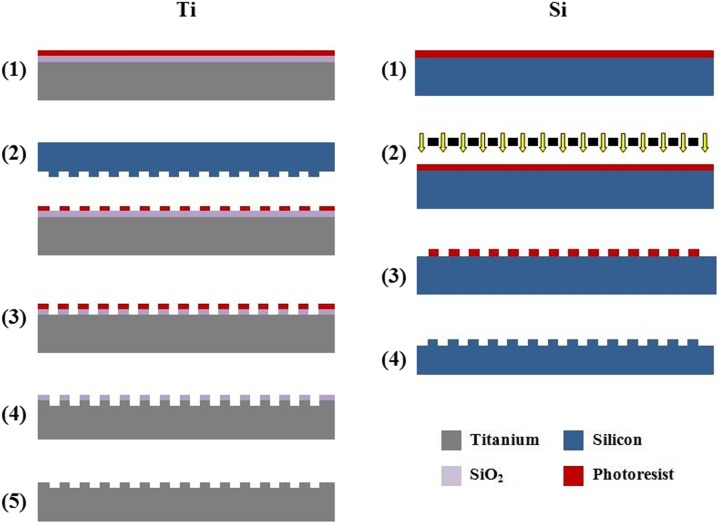
Fabrication processes for the patterned Ti (left) and Si (right) substrates. Patterned Ti substrate fabrication: 1) SiO_2_ deposition by PECVD, followed by PR application; 2) Lithographic patterning via thermal NIL with patterned Si imprint master; 3) Pattern transfer to SiO_2_ by F-based dry etching; 4) PR removal and Ti DRIE etch; and 5) Final SiO_2_ removal by F-based dry etching. Patterned Si substrate fabrication: 1) PR application; 2) Lithographic exposure via projection lithography; 3) PR development and O_2_ plasma descum; and 4) F-based dry etching and PR removal.

Figure 2 outlines the fabrication processes for the patterned Ti and Si substrates. In both cases, polished substrates were first subjected to a standard solvent cleaning procedure consisting of sequential sonication in acetone and isopropanol, followed by rinsing in deionized (DI) water, and drying with N_2_ gas.

For the patterned Ti substrate fabrication, polycrystalline, Grade 1, commercially-pure Ti substrates were used (99.6% Ti, 200 µm thickness; Tokyo Stainless Grinding). Following cleaning, an etch mask of 200 nm SiO_2_ was deposited using plasma enhanced chemical vapor deposition (PECVD) (VLR, Unaxis). Hexamethyldisilazane (HMDS) was then applied as an adhesion promoter, followed by photoresist (PR) spin-coating (mr-I 7020, Micro Resist Technology). Afterwards, the PR was patterned using a Si imprint master and thermal nanoimprint lithography (NIL) (NX2000, Nanonex). Oxygen-based dry etching was used to remove the residual PR layer at the base of the features after imprinting (E620-R&D, Panasonic Factory Solutions). This was followed by transfer of the PR patterns into the underlying SiO_2_ etch mask by fluorine-based dry etching (E620-R&D). The mask patterns were then transferred to a depth of 1.3 µm into the underlying substrate using a modified version of the Ti DRIE process optimized for nanoscale features. Finally, fluorine-based dry etching was used to remove the remaining etch mask.

For the patterned Si substrate fabrication, single crystal wafers were used (100 mm diameter, P/B doping, <1-0-0>, and 525±25 µm thickness; Silicon Quest International). Following cleaning, the wafers were dipped in buffered hydrofluoric acid, rinsed with DI, dried with N_2_, and dehydration baked. The wafers were then primed with HMDS, followed by PR spin-coating (AZ nLOF 5510, Clariant). After lithographic patterning using projection lithography (GCA Autostep 200 i-line Wafer Stepper, 3C Technical), the substrates were subjected to brief descuming by O_2_ plasma (PE-IIA, Technics) to ensure complete removal of PR residues that may remain at the base of the features after developing. The PR patterns were then transferred into the underlying Si substrate using fluorine–based dry etching (Plasmatherm SLR 770, Unaxis). Using this process, patterned Si substrates were produced for two purposes: 1) Substrates with grating depths of 1.3 µm were used for cell studies; and 2) Substrates with grating depths of 0.3 µm served as imprint masters in the fabrication of the patterned Ti substrates. For the latter, a coating of perflourodecyltricholorsilane (FDTS) was applied using molecular vapor deposition (MVD 100E, Applied Microstructures) to minimize resist adhesion during imprinting.

### Patterned Substrate Characterization

The fidelity and uniformity of the substrate patterning was characterized using scanning electron microscopy (SEM) (SUPRA 55, Leo). Imaging was performed at 5 kV accelerating voltage without need for application of conductive coatings on either substrate. Mean groove width, ridge width, and grating pitch for each sub-pattern were calculated based on measurements made at five different locations within each sub-pattern (i.e., center and four corners).

The groove depths and profiles in the patterned substrates were characterized via cross sectioning and SEM imaging. Focused ion beam (FIB) milling was used to cross section the Ti substrates (CrossBeam XB1540, Carl Zeiss Microscopy), while cleaving was used to cross section the Si substrates. The depths of the larger gratings of both substrates were corroborated using a surface profilometer with 12 m tip diameter stylus (Dektak 8, Veeco Metrology Group).

The surface roughness of the patterned substrates was characterized using atomic force microscopy (AFM) (Dimension 3100, Nanoscope IIIa, Veeco Metrology Group). Commercially-available silicon nitride tips were used (tip radius of curvature <10 nm, tip height = 14–16 µm, and spring constant = 1.2–6.4 N·m^−1^; Bruker AFM Probes). Imaging was performed in tapping mode with 1 Hz scan rate. Measurements were made within the middle of the ridge-tops for all gratings, well away from the ridge edges. For the 0.5 and 0.75 µm gratings, measurements were made over 0.20 µm×0.20 µm areas (i.e., 0.04 µm^2^ measurement area). For the 50 µm gratings and unpatterned sub-patterns, measurements were made over 0.53 µm×0.53 µm areas (i.e., 0.28 µm^2^ measurement area). Average roughness, root mean square roughness, and maximum roughness values for each sub-pattern were calculated based on measurements made at five different locations within each sub-pattern (i.e., center and four corners).

### Endothelial Cell Culture

EA926 cells, a HEC variant, were obtained from ATCC (Manassas, VA). Cells were cultured using Dulbecco’s modified Eagle’s medium (DMEM, Lonza) and were supplemented with 10% FBS (Gibco) and antibiotics (Primocin, Invivogen). Freshly passaged cells were maintained in a humidified air incubator (5% CO_2_, 37°C).

### Endothelial Cell Assays

Prior to all assays, the patterned substrates were subjected to standard solvent cleaning, followed by sterilization by autoclaving (121°C for 35 min) and overnight UV exposure. Trypsinized HECs were seeded at a density of 22,000 cells/cm^2^ on the patterned substrates, which were not subjected to any pre-treatment prior to seeding (i.e., neither oxygen plasma treatment, nor pre-incubation with fibronectin, collagen, BSA, etc.). The cell-seeded substrates were cultured for various durations (30 min, 1 d, and 5 d), after which non-adherent cells were removed by rinsing twice in phosphate buffered solution (PBS). Cells that remained on the surface were visualized by fluorescent staining of nuclei to evaluate adhesion and proliferation response (Hoechst 33342, Life Technologies), or the cellular membrane to evaluate substrate coverage (Rhodamine 123, Life Technologies). Live/dead assays were also performed on surface-adhered cells using propidium iodide and 4′,6-diamidino-2-phenylindole (Life Technologies). Mean cell densities for each sub-pattern were calculated based on measurements made at five different locations within each sub-pattern (i.e., center and four corners).

### Immunostaining

For morphological and cytoskeletal architecture studies, non-adherent HECs were removed by PBS rinsing after prescribed culture durations. Remaining HECs on the patterned substrates were fixed with 4% paraformaldehyde for 15 min, permeabilized with 0.2% Triton X-100, blocked with 1 mg/ml BSA for 10 min, rinsed with PBS, and stained for 10 min with Alexa Fluor 488 phalloidin (Life Technologies) for F-actin, and Hoechst 33342 (Life Technologies) for nuclei. The elongation and orientation of HECs on the patterned substrates were determined based on measurements made on immunostained cells after 5 d culture. Using ImageJ, elongation was calculated as the ratio of major to minor cell axis lengths (as defined by the actin microfilament network), while orientation was characterized as the angular deviation between the cell major axis and the grating axis. Angular deviation for HECs on the unpatterned sub-patterns was determined relative to an arbitrary reference axis whose orientation was held fixed for each field of view the measurements were made over. Means were calculated based on measurements made at five different locations within each sub-pattern (i.e., center and four corners).

For phenotype and function studies, the expression of two EC markers, von Willebrand Factor (vWF) and vascular cell adhesion molecule-1 (VCAM-1), was characterized. vWF maintains homeostasis through binding to FVIII, platelet surface glycoproteins, and constituents of connective tissue. It also initiates platelet aggregation via binding to exposed structures of injured vessel walls at high arterial shear rates. Furthermore, it is thought to assist during platelet aggregation by bridging adjacent platelets at high shear rates. The function of vWF is strongly shear rate dependent, whereas fluid dynamic conditions, as well as mechanical forces, are crucial for the conformational transition of VWF to develop its interaction with endothelial matrix proteins, as well as platelets, in case of vessel injury [30], [31]. Essentially, vWF is considered an anti-thrombotic biomarker [32]. VCAM-1 plays an important role in both immune responses and in recruitment of lymphocytes, monocytes, leukocyte adhesion to sites of inflammation [33]. It appears to function as a leukocyte-endothelial cell migration molecule [34]. Because of this, VCAM-1 is recognized as a significant biomarker of endothelial dysfunction [35], [36].

HECs were seeded at a density of 50,000 cells/cm^2^ and cultured for 1 day. Substrates were then washed with PBS and adherent HECs were fixed and permeabilized in −20°C methanol for 20 min. Substrates were then washed in PBS, incubated with blocking buffer (4 g BSA+80 mL PBS+150 µL Triton×100) for 1 h, and incubated with 1∶400 of rabbit polyclonal anti-vWF/VCAM-1 in antibody dilution buffer (4 g BSA+40 mL PBS+120 µL Triton×100) overnight at 4°C. Substrates were then rinsed in PBS and incubated with 1∶1000 Texas Red 598 donkey anti-rabbit secondary antibody for 1 h at 25°C. Finally, nuclei were cross-stained using Hoechst 33342. Using ImageJ, protein expression per cell area was quantified by measuring the average fluorescence signal intensity within a cell and dividing this value by the area of the cell. Means were calculated based on measurements made on at least 10 cells per sub-pattern.

### Fluorescent Imaging

Fluorescent imaging of HECs on the patterned substrates was performed using a fully apochromatic corrected stereomicroscope with 12.5∶1 zoom (M125, Leica). Images were acquired with a 10X objective lens, binning of 4×4, gain of 8.0, and brightness of 1.2 was used for image acquisition. Stained cells were imaged using a Leica SP5 confocal microscope. Spot Imaging Software and Leica SP5 LAS Software were used for image acquisition, and images were processed using ImageJ (v1.46, NIH).

### Scanning Electron Microscopy of Cell-seeded Substrates

SEM imaging of HECs on the patterned substrates was also performed. Prior to imaging, cells were washed with PBS, fixed with 4% glutaraldehyde, and post-fixed with 0.5% OsO_4_ for 1 h each. They were then dehydrated through a graded series of alcohols and dried in a critical point dryer (CPD 030, Balzer). Imaging was performed at 5 kV accelerating voltage without need for conductive coatings on either substrate type.

### Statistical Analyses

All cell studies were repeated in triplicate. Statistical analyses were performed using single-factor ANOVA and commercially-available software packages (Excel, Microsoft; and SigmaPlot 5.0, Systat Software).

## Results

### Patterned Substrate Characterization

**Figure 3:**
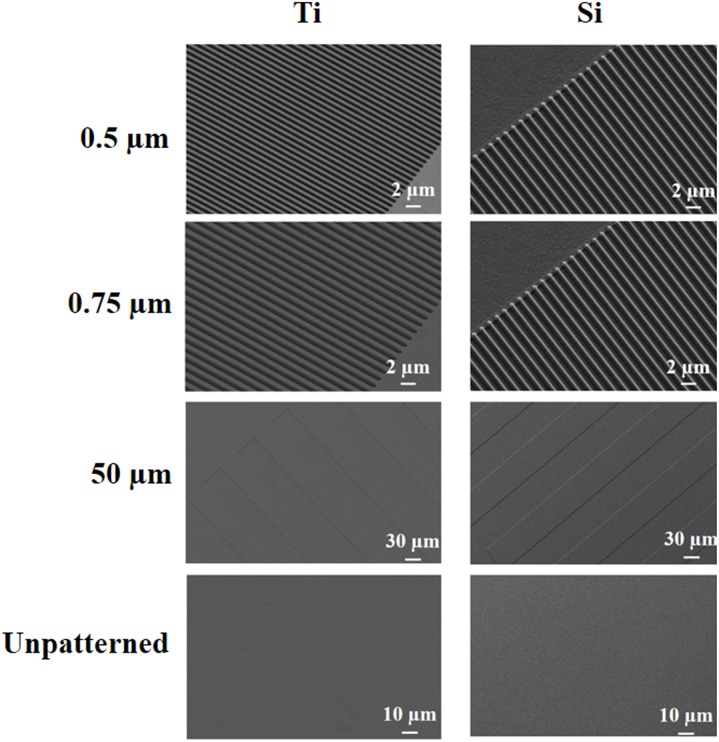
Scanning electron microscope micrographs of selected patterned Ti (left) and Si (right) substrates, as well as unpatterned control surfaces.

Figure 3 shows representative SEM micrographs of selected patterned Ti and Si substrates. Precisely defined and highly uniform patterning is observed on both substrate materials. This is further corroborated by the excellent agreement between the expected and measured grating groove widths, ridge widths, and pitches for selected sub-patterns reported in Table 1. Similar agreement was observed for the other sub-patterns (data not shown). As discussed earlier, the Ti DRIE process is the only technique capable of producing such diminutive and precisely-defined features within bulk Ti substrates.

**Table 1:** Scanning electron microscope measurements of selected sub-pattern feature dimensions on patterned Ti and Si substrates, including grating groove width, ridge width, and pitch (i.e. groove width + ridge width).

	Ti			Si		
Feature Size	0.5 µm	0.75 µm	50 µm	0.5 µm	0.75 µm	50 µm
**Groove (**µ**m)**	0.498±0.003	0.748±0.000	50.0±0.0	0.501±0.002	0.747±0.000	50.0±0.0
**Ridge (**µ**m)**	0.502±0.003	0.748±0.000	50.0±0.0	0.501±0.002	0.751±0.006	50.0±0.0
**Pitch (**µ**m)**	1.000±0.000	1.500±0.004	100.0±0.0	1.000±0.001	1.510±0.000	100.0±0.1

Data = mean ± standard deviation (n = 5).

**Figure 4:**
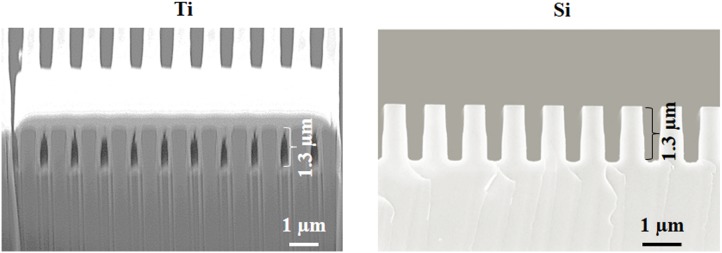
Scanning electron microscope micrographs of 0.5 µm grating sub-pattern cross sections from patterned Ti (left) and Si (right) substrates. The Ti grating was imaged in a tilted orientation, while the Si grating was imaged without tilt. The bright layer covering the upper foreground surface of the Ti grating is a Pt film deposited prior to focused ion beam milling. This film protected the underlying structures from sputtering-induced faceting during milling. The vertically-oriented contrast variations observed beneath the Ti grating are “curtain effect” artifacts produced by differential sputtering during milling.

Figure 4 shows cross section SEM micrographs of the 0.5 µm gratings on the patterned Ti and Si substrates. As can be seen, nearly identical gratings have been produced in both substrates. Moreover, groove profiles are observed to be nearly rectangular. Finally, identical groove depths of 1.3 µm are achieved for both substrates. Results from surface profilometry measurements of wider grooves elsewhere on the patterned substrates returned similar depths (data not shown), thus indicating a uniform groove depth across all sub-patterns on the Ti and Si substrates.

Table 2 reports AFM-based surface roughness measurements for the grating ridge-tops of selected patterned Ti and Si substrates. Average roughness, R_a_, represents the average height of the roughness irregularities, while root mean square roughness, R_sq_, is more sensitive to low and high points, and maximum roughness, R_max_, reports the largest aberrations. In all sub-patterns, R_a_ and R_sq_ are extremely small (i.e., ≤2 nm), thus suggesting that this should minimally influence cellular response.

**Table 2:** Atomic force microscope measurements of selected sub-pattern ridge-top surface roughness on patterned Ti and Si substrates, including average roughness (R_a_), root mean square roughness (R_sq_), and maximum roughness (R_max_).

	Ti				Si			
Feature Size	0.5 µm	0.75 µm	50 µm	unpatterned	0.5 µm	0.75 µm	50 µm	unpatterned
**R_a_ (nm)**	0.88±0.19	0.62±0.05	0.43±0.04	0.56±0.03	0.39±0.01	0.60±0.07	0.30±0.03	1.60±0.15
**R_sq_ (nm)**	1.15±0.18	0.77±0.28	0.56±0.05	0.74±0.06	0.52±0.01	0.91±0.22	0.40±0.07	2.01±0.19
**R_max_ (nm)**	10.48±4.23	5.06±0.41	7.89±2.19	7.05±1.08	5.30±1.58	11.56±2.38	5.21±1.63	14.23±1.27

Data = mean ± standard deviation (n = 5).

### Endothelial Cell Adhesion and Proliferation

**Figure 5:**
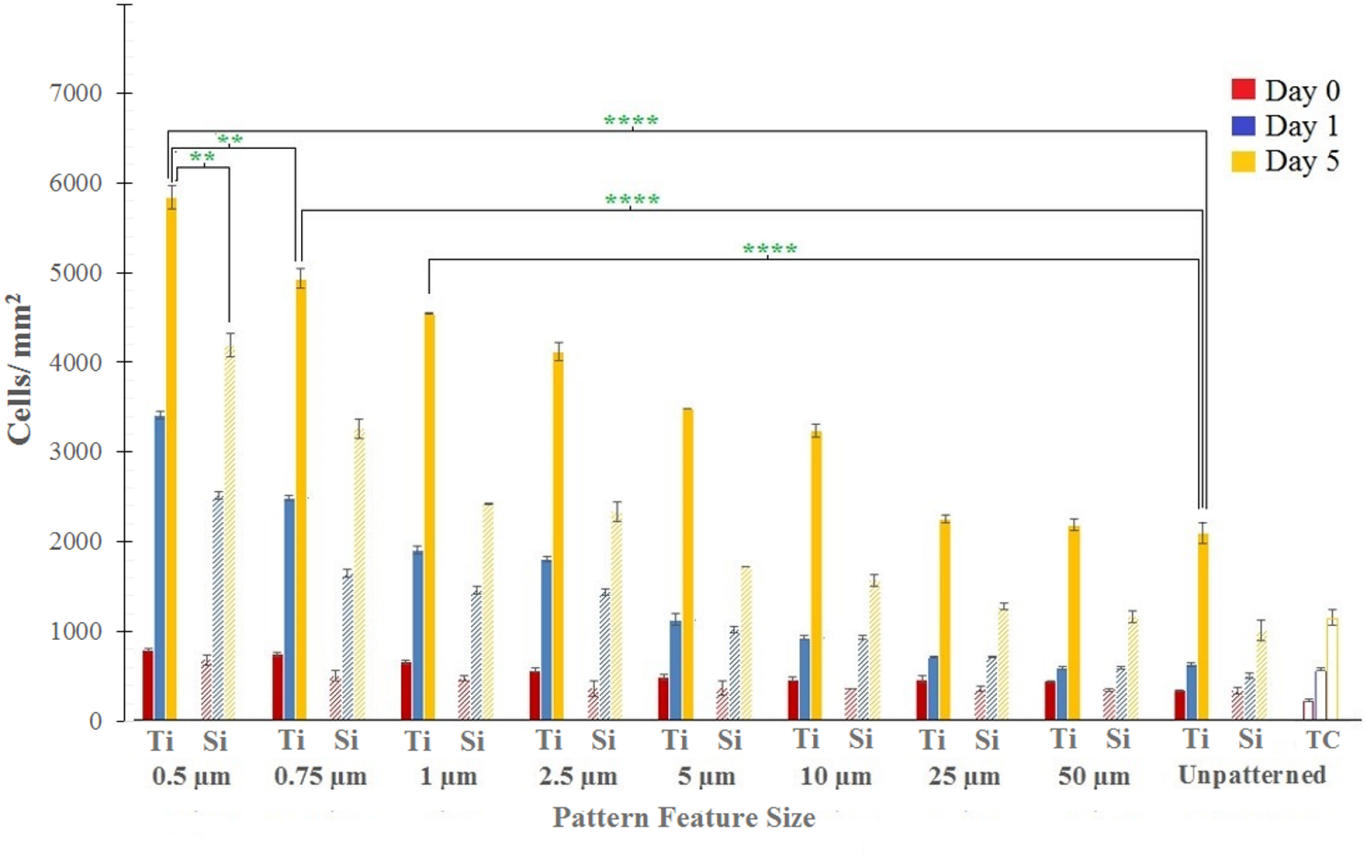
Human endothelial cell densities on patterned Ti and Si substrates and tissue culture plastic at varying time points ranging from 30 min (i.e. 0 days) to 5 days. Data = mean ± SEM (**p = 0.01, ****p = 0.0001; unpaired samples T-test, n = 5).

Figure 5 shows HEC densities at various time points on the patterned Ti and Si substrates. At the 0 d time point (i.e. 30 min), we observe a trend of increasing adhesion with decreasing feature size on the patterned Ti substrates, and response on the patterned Ti surfaces is greater than the unpatterned Ti control, e.g., HEC densities on the 0.5 µm Ti gratings are 2.32 times greater than unpatterned Ti. For the patterned Si substrates, similar size-dependent response is observed, e.g., HEC densities on the 0.5 µm Si gratings are 2 times greater than unpatterned Si. However, adhesion on patterned Si is generally lower than on comparably patterned Ti, e.g., HEC densities on the 0.5 µm Si gratings are 14% lower than on 0.5 µm Ti gratings. Finally, adhesion on both patterned and unpatterned Ti and Si is greater than the tissue culture plastic control.

At latter time points (i.e., 1 d and onwards), Figure 5 shows that HEC proliferation increases with decreasing feature size on the patterned Ti substrates, and response on patterned Ti surfaces is greater than the unpatterned Ti control, e.g., at 5 d, HEC densities on the 0.5 µm Ti gratings are 2.79 times greater than unpatterned Ti. A similar trend for the patterned Si substrates is also seen, e.g., at 5 d, HEC densities on the 0.5 µm Si gratings are 4.14 times greater than unpatterned Si. However, proliferation on patterned Si is generally lower than on patterned Ti at comparable time points and feature sizes, e.g., at 5 d, HEC densities on the 0.5 µm Si gratings are 28.26% lower than on the 0.5 µm Ti gratings. Finally, proliferation on patterned Ti and Si is generally greater than the tissue culture plastic control. For all time points and sub-patterns, live/dead assays indicate a maximum of 0.1% of dead cells on both Ti and Si substrates, thus confirming the viability of the adhered cells.

### Endothelial Cell Morphology and Cytoskeletal Architecture

**Figure 6:**
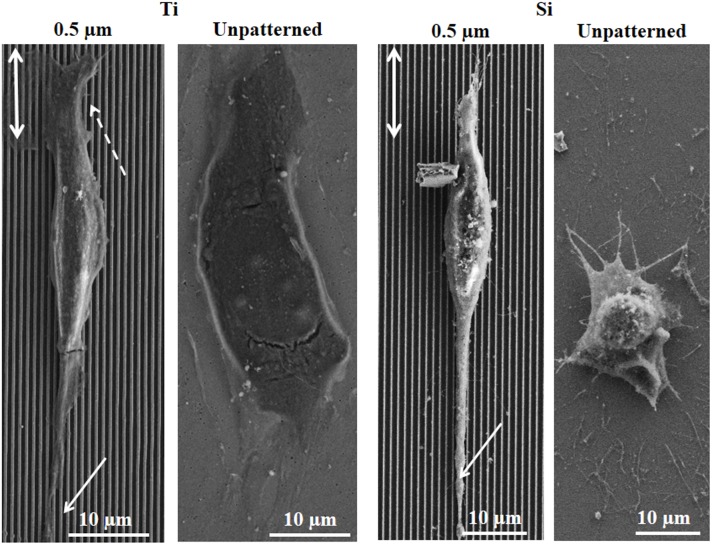
Scanning electron microscope micrographs of human endothelial cells after 1 day culture on 0.5 µm gratings and unpatterned sub-patterns of Ti (left) and Si (right) substrates. Arrows indicate filopodia and lamellopodia.

Figure 6 shows SEM micrographs of HECs on 0.5 µm gratings and unpatterned sub-patterns of both substrate materials after 1 d. Significant cellular elongation is observed on the Ti grating and alignment along the grating axis is clear. For the Si grating, significant cellular elongation is also seen, as is alignment with the grating axis. However, more favorable cellular morphology occurs on the Ti grating relative to the Si grating, as evidenced by greater flattening and spreading. Greater spreading is also seen on the unpatterned Ti relative to unpatterned Si surfaces.

**Figure 7:**
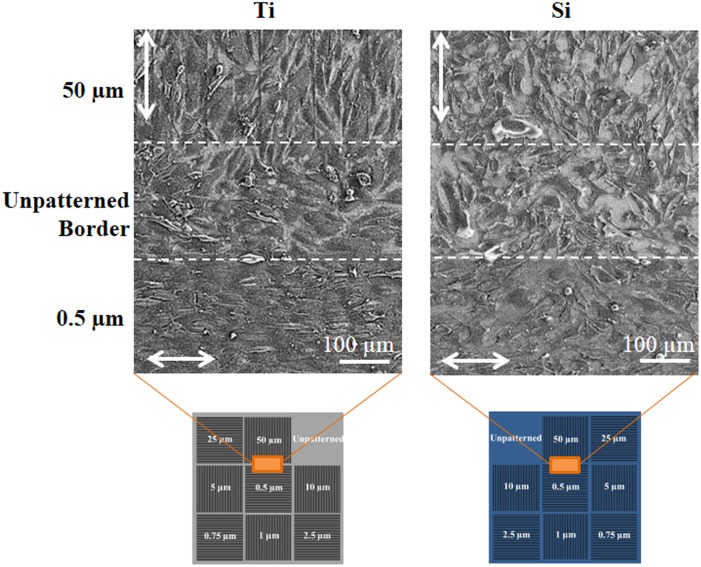
Scanning electron microscope micrographs of human endothelial cells after 5 day culture on patterned Ti (left) and Si (right) substrates. Each micrograph depicts a single field of view located at the boundary between the 0.5 µm and 50 µm grating sub-patterns, as illustrated in the schematics below each micrograph. Dotted lines indicate boundaries of the sub-patterns and double arrows indicate grating directions.

Similar trends are observed at latter time points, as illustrated in Figure 7, which shows SEM micrographs of HECs on patterned Ti and Si substrates after 5 d. These images were taken at the boundary between the 0.5 µm and 50 µm sub-patterns, which are separated by a 200 µm wide unpatterned region, and are orthogonally oriented with respect to one another. Cellular elongation and alignment are again seen on the 0.5 µm gratings of both materials, with more favorable morphology and greater coverage on the 0.5 µm Ti grating. Moreover, the Ti micrograph clearly illustrates the spatial specificity of HEC response to the 0.5 µm Ti grating, as evidenced by the decreasing cell alignment and density with distance from the boundary of the 0.5 µm sub-pattern.

**Figure 8:**
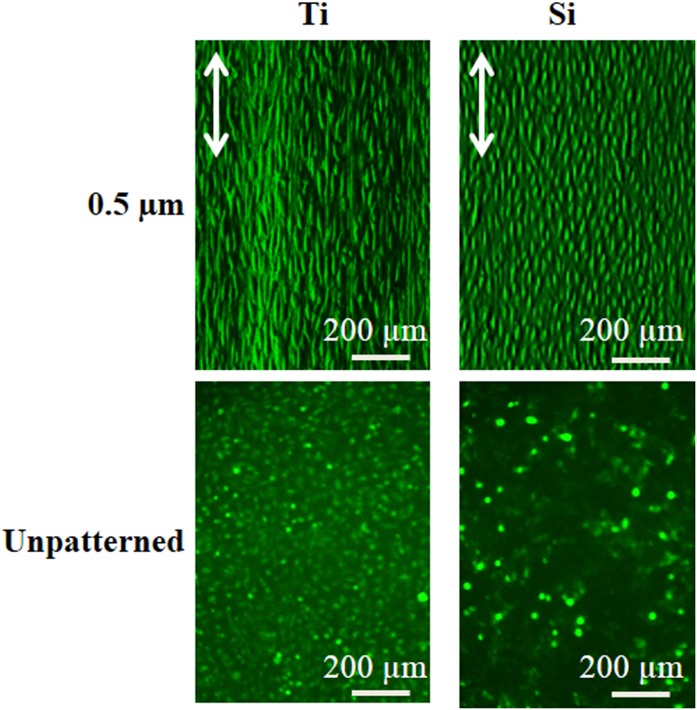
Fluorescent micrographs of human endothelial cells after 5 day culture on 0.5 µm gratings and unpatterned sub-patterns of Ti (left) and Si (right) substrates. Cells were stained with Rhodamine 123. Double arrows indicate grating direction.

Further evidence of the influence of sub-micrometer patterning and substrate material is provided by Figure 8, which shows fluorescence micrographs of HECs on 0.5 µm gratings and unpatterned sub-patterns of both substrate materials after 5 d. Strong elongation and alignment are observed on the Ti grating. Moreover, a nearly confluent HEC layer is observed on the Ti grating, while sparser coverage is seen on the unpatterned Ti. For the Si grating, increased elongation and alignment are also observed relative to the unpatterned Si control. However, HEC coverage on the Si grating is lower than the comparable Ti grating.

**Figure 9:**
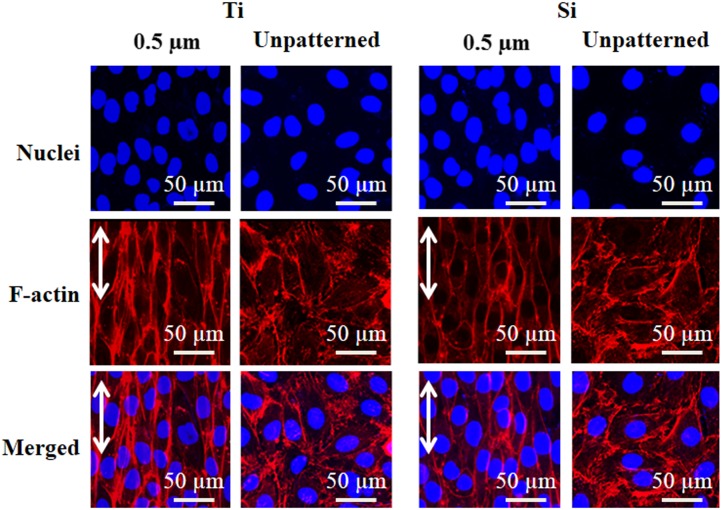
Confocal micrographs of human endothelial cells after 5 day culture on 0.5 µm gratings and unpatterned sub-patterns of Ti (left) and Si (right) substrates. Cells were immunostained using phalloidin (red) for cytoskeletal protein F-actin, and Hoechst 33342 (blue) for nuclei. Double arrows indicate grating direction.

The influence of sub-micrometer patterning and substrate material on cytoskeletal architecture is illustrated in Figure 9, which shows HECs stained for nuclei and F-actin on 0.5 µm gratings and unpatterned sub-patterns of both substrate materials after 5 d. Strong cytoskeletal alignment is evident on the gratings of both materials, which is corroborated by the increasing cellular elongation ratios and decreasing angular deviations with decreasing feature size reported in Table 3. However, the microfilament network on the 0.5 µm Ti grating is observed to be more robust relative to the 0.5 µm Si grating, and greater elongation is seen on the Ti grating. Cytoskeletal alignment is not observed on the unpatterned controls for either substrate. This is further corroborated by the measured ∼45° mean angular deviation on the unpatterned controls for either substrate, which is indicative of randomized cell orientation.

**Table 3:** Confocal microscopy measurements of human endothelial cell elongation ratio and angular deviation (from the grating axis) after 5 day culture on patterned Ti and Si substrates.

	Ti				Si			
FeatureSize	0.5 µm	0.75 µm	50 µm	unpatterned	0.5 µm	0.75 µm	50 µm	unpatterned
**Elongation** **Ratio**	5.62±1.23	4.79±0.93	3.49±0.88	2.89±0.85	3.52±0.60	2.03±0.34	2.37±0.79	2.87±0.82
**Angular** **Deviation** **(degrees)**	4.66±3.47	10.30±4.34	45.37±3.02	45.35±23.01	3.33±2.70	17.79±14.52	42.1±18.47	47.63±45.95

Angular deviation on unpatterned sub-patterns was determined relative to an arbitrary reference axis that was held fixed for each field of view. Data = mean ± standard deviation (n = 5).

### Endothelial Cell Function

**Figure 10:**
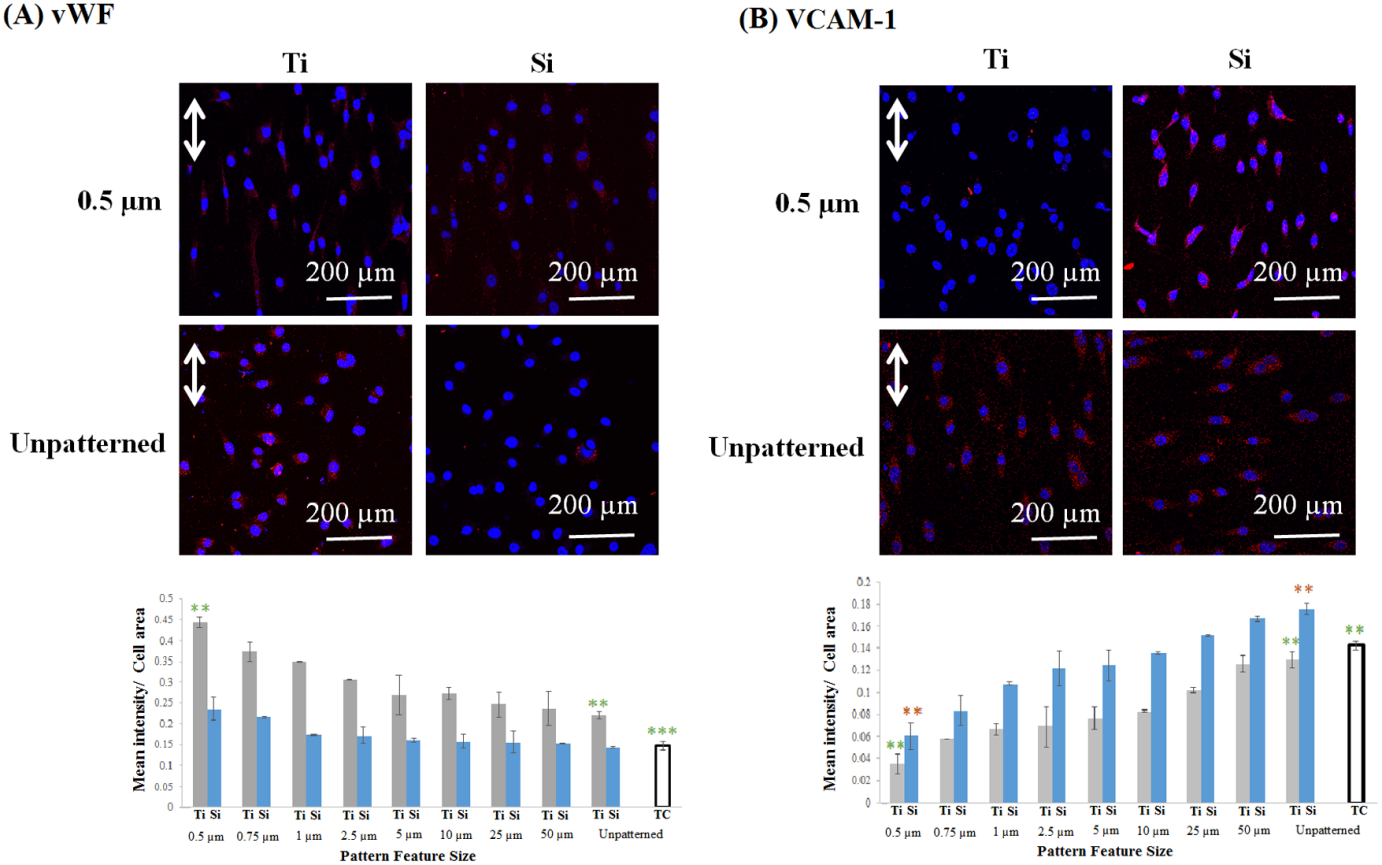
Confocal micrographs and quantification of human endothelial cell protein expression (red) after 1 day culture on 0.5 µm gratings and unpatterned sub-patterns of Ti and Si substrates, and tissue culture plastic: A) vWF; and B) VCAM-1. Nuclei (blue) were cross-stained using Hoechst 33342. Double arrows indicate grating direction. Data = mean ± SEM (**p = 0.01, ***p = 0.001; unpaired samples T-test, n = 10).

Figure 10 shows results for expression of two important EC markers, vWF and VCAM-1, on the patterned Ti and Si substrates. As discussed earlier, vWF is a functional marker expressed by ECs that plays a key role in homeostasis, whereas VCAM-1 is a marker for inflammation.

As shown in Fig. 10A, vWF is uniformly distributed within the cytoplasm of HECs on the 0.5 µm gratings of both Ti and Si, but is more confined to the perinuclear regions on the unpatterned controls. Moreover, quantitative measurements show increasing expression with decreasing feature size on both Ti and Si patterned substrates, and response on the patterned surfaces is greater than their respective unpatterned controls, e.g., expression on the 0.5 µm Ti gratings is 2 times greater than unpatterned Ti. However, expression on patterned Si is considerably lower than on comparably patterned Ti, e.g., expression on the 0.5 µm Si gratings is 46% lower than on 0.5 µm Ti gratings. Finally, expression on both patterned and unpatterned Ti and Si is generally greater than the tissue culture plastic control.

Figure 10B shows that expression of VCAM-1 is also feature size-dependent, although in a converse manner, with expression largely confined to the perinuclear regions in the 0.5 µm gratings of both Ti and Si, but more widely distributed on the unpatterned controls. Moreover, quantitative measurements show decreasing expression with decreasing feature size on both Ti and Si patterned substrates, and expression patterned surfaces is lower than the unpatterned controls, e.g., expression on the 0.5 µm Ti gratings is 3.6 times lower than unpatterned Ti. However, expression on patterned Si is higher than on comparably patterned Ti, e.g., expression on the 0.5 µm Si gratings is 41% higher than on 0.5 µm Ti gratings. Finally, expression on sub-micrometer patterned Ti and Si is lower than the tissue culture plastic control.

## Discussion

The data presented herein demonstrate that EC response is enhanced with decreasing feature size on patterned Ti substrates down to 0.5 µm; specifically, decreasing feature sizes are shown to promote greater adhesion, proliferation, and elongation, as well as a more athero-resistant phenotype *in* *vitro*, which therefore suggests promise for facilitating the reestablishment of a functional endothelium *in* *vivo*. Moreover, the data show that patterning of Si substrates also elicits favorable trending in EC response with decreasing feature size, albeit to a lesser extent than comparable Ti gratings. This therefore suggests that while topographical cueing can be used to promote enhanced EC response on both materials, their differing surface chemistries affect the ultimate magnitude of cellular response.

### Endothelial Cell Response as a Function of Substrate Material and Topography

While exploration of the mechanisms underlying the differential EC response observed on comparably patterned Ti and Si substrates is beyond the scope of the current study, one potential explanation could lie in the differing stiffnesses of these substrates. Studies have shown that ECs, like many adherent cells, are affected by variation in substrate stiffness. Typical responses include migration toward stiffer regions, as well as increased adhesion and spreading with increasing substrate stiffness [37]–[40]. However, our observations (e.g., Figure 6) show reduced spreading on the stiffer Si substrates (E_Ti_ = 105 GPa [41] & E_Si_ = 130–188 GPa [42]). Moreover, the stiffnesses of the current substrates are likely to be well-beyond the threshold for significant differential response, since their moduli greatly exceed those of the polymeric substrates used in previously reported studies (0.1 kPa–2.5 MPa). Consequently, this suggests against substrate stiffness as a potential explanation in the current study.

An alternative, and perhaps more plausible explanation for the observed differential response may lie in the differing adhesive ligand presentation on these materials. It is well known that adhesive ligands (e.g., R-G-D) presented by surface-adsorbed plasma proteins (e.g., fibrinogen and fibronectin) act as binding sites for transmembrane integrins. Moreover, it is well known that the specific presentation of these ligands, in turn, depends upon the physicochemical properties of the substrate [7]. Finally, it has been recently shown that EC adhesion and spreading typically increase with increasing ligand density until saturation [43], [44]. Consequently, this suggests that adhesive ligand density and/or presentation is more favorable on Ti compared to Si (or more specifically, on the oxide surfaces presented thereon [45]), as evidenced by the increase in cell density (Figure 5), spreading (Figure 6), and athero-resistant signalling modulation (Figure 10) observed on Ti relative to comparably patterned Si.

Regarding topography specifically, it is well-known that micro- to nano-scale topography can independently affect the amount, spatial distribution, and conformation of adsorbed proteins, as well as the composition of the adsorbed protein layer that forms during exposure to complex biological fluids [46]. Moreover, it has been recently shown that fibronectin adsorption is increased considerably on patterned Si substrates with deep submicrometer scale gratings when compared to planar controls [47], [48], as is the degree of native folding [48]. This suggests that the enhanced EC responses observed in the current study may arise from increasing protein adsorption with decreasing features sizes, which provides increased protein-protein interactions that help stabilize native conformations against denaturization by protein-substrate interactions. However, the considerable differences in grating depth (i.e., 0.09 µm in [47], [48] vs. 1.3 µm in the current study) and profile (i.e., sinusoidal in [48] vs. rectangular in the current study) demonstrate the need for further studies to quantify protein adsorption and conformation on the current patterned Ti and Si substrates specifically.

### Comparison to Previous Studies

Although the current study is the first to explore differential EC response on micrometer to sub-micrometer patterned Ti and Si substrates, it is instructive to compare our observations to those reported by others for polymeric substrates patterned with gratings of similar feature size. As in the current study, cellular alignment and elongation along the grating axis is reported in nearly all studies with features smaller than the dimensions of typical ECs (i.e., features <20 µm) [9]–[15], [18]–[25]. However, while increasing proliferation with decreasing groove width is observed in the current study, the opposite [13], [19], [25] or minimal differential response [11] has been reported in studies with patterned polymers. Moreover, while decreasing features sizes are shown to upregulate vWF and downregulate VCAM-1 expression in this study, minimal differential expression has been reported by others [13], [23]. These differences may arise from a number of factors, including, among others: a) the relatively shallow nature of the grooves in those earlier studies (i.e., groove depth ≤1 µm vs. 1.3 µm for the current study); b) the different cell types used (i.e., human umbilical vein ECs, human endothelial progenitor cells, & bovine aortic ECs vs. HECs in the current study); c) the differing substrate surface chemistries and mechanical properties (e.g., polyurethane, cylic olefin copolymer, polydimethylsiloxane, etc. vs. Ti & Si in the current study); and/or d) the absence of substrate pre-treatment prior to cell seeding in the current study (i.e., neither oxygen plasma treatment, nor pre-incubation with fibronectin, collagen, BSA, etc. were used in the current study).

### Implications for Ti- and Si-based Implantable Microdevices

The favorable trending of EC response with decreasing feature size in the current study suggests promise for use of patterning as a means for improving the safety, efficacy, and performance of novel Ti- and Si-based implantable microdevices.

For example, within the context of vascular stents, the observed enhancement of proliferation and athero-resistant phenotype *in* *vitro* suggests potential for use of patterning to accelerate endothelialization and healing *in* *vivo*. This could therefore provide means for addressing a lingering safety concern associated with current drug-eluting stents, namely late-stent thrombosis resulting from delayed healing [49]. Indeed, first evidence demonstrating this potential *in* *vivo* was recently reported by Sprague *et al*., who showed that endothelialization was accelerated on stents patterned with 15 µm groove width gratings [50]. However, since the current study demonstrates favorable trending of EC response with decreasing feature size into the sub-micrometer realm, this suggests potential for even further improvement. We have recently demonstrated the fabrication of the first sub-micrometer patterned stents that will eventually provide capability for evaluating this potential *in* *vivo* [26].

It is also conceivable that patterning-induced enhancement of EC response could provide means for facilitating greater neovascularization on Si-based wirelessly-controlled implantable drug delivery microchips [27]. As reported by Bettinger *et al*., sub-micrometer patterning of a polymeric substrate can promote the formation of EC-based supercellular band structures that facilitate the subsequent formation of capillary tubes roughly aligned with the grating axis [13]. As such, potential may exist for using patterning to guide capillary tube formation towards the reservoir openings on the surface of drug delivery microchips. Although further studies are required to validate this conjecture, this suggests potential for facilitating the establishment of a direct connection to the surrounding vasculature for applications where rapid systemic delivery is required. Moreover, the observation in the current study of greater EC response on patterned Ti relative to patterned Si suggests the potential superiority of Ti for such devices.

## Conclusions

We have demonstrated the fabrication of precisely-defined, grating-based patterns on bulk Ti and Si substrates with groove widths ranging from 0.5–50 µm. *In vitro* studies evaluating HEC adhesion, proliferation, morphology, and functionality on these substrates have shown favorable trending of cellular response with decreasing feature size on the patterned Ti substrates. These studies have also shown that patterning enhances HEC response on Si substrates, although to a lesser extent than comparably patterned Ti. Collectively, these results suggest promise for using sub-micrometer topographic patterning to enhance the safety, efficacy, and performance of implantable microdevices based on these substrate materials. Moreover, these results particularly highlight the potential superiority of sub-micrometer patterned Ti for such applications, thus motivating further studies in this regard.
